# Novel SARS-CoV-2 Variants in Italy: The Role of Veterinary Public Health Institutes

**DOI:** 10.3390/v13040549

**Published:** 2021-03-25

**Authors:** Alessio Lorusso, Paolo Calistri, Giovanni Savini, Daniela Morelli, Lucio Ambrosij, Giacomo Migliorati, Nicola D’Alterio

**Affiliations:** Istituto Zooprofilattico Sperimentale dell’Abruzzo e del Molise ‘G. Caporale’, 64100 Teramo, Italy; p.calistri@izs.it (P.C.); g.savini@izs.it (G.S.); d.morelli@izs.it (D.M.); l.ambrosij@izs.it (L.A.); g.migliorati@izs.it (G.M.); n.dalterio@izs.it (N.D.)

**Keywords:** SARS-CoV-2, diagnosis, variants, whole genome sequencing

## Abstract

Novel SARS-CoV-2 variants with potential impacts on diagnostics, antivirals, and vaccines are spreading in Italy. In this editorial, we highlight the role that veterinary public health institutes may have in this global crisis, as their expertise in genomic/antigenic surveillance and animal studies are crucial to tackle SARS-CoV-2 pandemic.

## Introduction

Italy’s health care system is a regionally-based national health service (Servizio Sanitario Nazionale (SSN)) that provides universal coverage free of charge at the point of service. The national level is responsible for ensuring the general objectives and fundamental principles of the national health care system.

In Italy, the Departments of Health Prevention, organized into nearly 100 Local Health Units (ASL), are also in charge of implementing all SARS-CoV-2 surveillance and tracing activities. Surveillance is mainly based on respiratory swab testing by real time RT-PCR in approved public laboratories, on all individuals with clinical signs compatible with COVID-19 (persisting fever, dyspnea, anosmia, etc.), and those in strict contact with confirmed cases in the 14 days before clinical onset. In addition, mass drive-through testing initiatives, with the use of antigenic rapid tests, are organized in ports, airports, main train and bus stations, major cities, and towns experiencing a significant increase of cases. People with positive results to antigenic rapid tests are then subjected to a following swab collection for molecular testing. In addition, periodic testing is performed on at-risk worker categories, such as health and laboratory personnel. In turn, sequencing and genome analysis activities directly from infected swab samples are still far from optimal, and they are performed on a voluntary basis by equipped laboratories scattered all over the Italian territory. However, out of a total number of 834,274 sequences available on GISAID (gisaid.org) up to 22 March 2021, only 13,769 originated from Italy, with huge differences between regions.

In December 2020, four nasopharyngeal swab samples (NSSs) collected from patients in Guardiagrele (Chieti province, Abruzzo Region, Italy) were positive for SARS-CoV-2 RNA, showing a readout pattern characterized by the detection of the replicase and N genes, but negative for the S gene. Such a readout is suggestive of the VOC 202012/01 (B.1.1.7 lineage), which emerged with a large number of mutations on 12 September 2020 in the south of the UK [1]. Since then, it became the predominant variant in the UK, linked to a significant increase of COVID-19 transmissibility and upsurge of incidence and hospitalizations in the second half of December 2020 [2]. In the meantime, additional samples from Guardiagrele and other municipalities of Chieti province showed the same readout pattern. Eventually, the Istituto Zooprofilattico Sperimentale dell’ Abruzzo e Molise (IZSAM), a veterinary public health institute, which operates in the Abruzzo and Molise Regions as a technical and scientific arm of the Italian Ministry of Health, released up to 22 March 2021 with GISAID 910 sequences of the B.1.1.7 lineage originating from different Italian regions, including Abruzzo, Molise, and Umbria.

Three NSSs collected on 18 January 2021 from travelers under quarantine, who returned from Brazil to a small village located in L’Aquila province (Abruzzo Region), tested positive for SARS-CoV-2 RNA, and were immediately sequenced. In all samples, the P.1 lineage, a new variant first detected with very high attack rates in December 2020 in Manaus [3] (Amazonas state, Brazil), was evidenced. This lineage contains a unique constellation of mutations including E484K, K417T, and N501Y in the S protein. Such mutations could have a role in evading the host immune response [4]. On 26 January, the three P.1 sequences were released with GISAID. As a consequence, the Italian Ministry of Health has ordered a ban on direct flights to Italy from Brazil [5].

While most emerging mutations of SARS-CoV-2 do not have an apparent impact on viral spread, some mutations or combinations of mutations may provide the virus with a selective advantage, such as increased transmissibility, as apparently testified by the emergence and spread of the B.1.1.7 and P.1 lineages in some countries, but also the ability to evade the host immune response, with potential impacts on countermeasures (e.g., diagnostics, antivirals, and vaccines). Hence, a global effort to sequence and share SARS-CoV-2 genomes is crucial to track mutations, as well as to investigate viral evolution and transmission dynamics.

Sequencing activities must be coupled with in vitro and in vivo experimental studies in order to disentangle the role of specific mutations and the impact of these on acquired SARS-CoV-2 immunity. In Italy, veterinary public health institutes such IZSAM could play a relevant role in these tasks, as they are equipped with large infrastructures for in vivo and in vitro studies, antigenic cartography, genomic analysis, and storage of sequence data routinely used in animal health and food security emergences.

Although SARS-CoV-2 likely originated from an animal reservoir [6,7], the exact mechanisms of emergence, the host species involved, and the risk to domestic and agricultural animals are largely unknown. Some domestic animal species, including cats, ferrets, and notably, minks, have been demonstrated to be susceptible to SARS-CoV-2 infection, while others, such as pigs and chickens, are not. In this perspective, SARS-CoV-2 surveillance in animals [8] is a fundamental task, ruled by veterinary public health institutes such IZSAM, to characterize potential novel viral variants with hazard to humans [9,10,11,12].
